# Experimental Investigation into Tensile Mechanical Properties of the Unidirectional Flax Fibre–Reinforced Vitrimer Composite—Seeking Sustainable Opportunities for the Automotive Industry

**DOI:** 10.3390/ma19132687

**Published:** 2026-06-23

**Authors:** Milan M. Janković, Igor M. Balać, Mihajlo D. Popović, Miloš D. Pjević, Robert Bjekovic

**Affiliations:** 1 Faculty of Mechanical Engineering, University of Belgrade, 11120 Belgrade, Serbia; ibalac@mas.bg.ac.rs (I.M.B.); mpopovic@mas.bg.ac.rs (M.D.P.); mpjevic@mas.bg.ac.rs (M.D.P.); 2 Faculty of Mechanical Engineering, University of Applied Sciences Ravensburg-Weingarten, 88250 Weingarten, Germany; robert.bjekovic@rwu.de

**Keywords:** tensile mechanical properties, material, polymer, flax/vitrimer composite, fabrication, experimental, sustainable, automotive

## Abstract

Emerging sustainability demands and calls for lowering materials’ environmental impact have directed authors to examine a class of polymers characterised as covalent adaptable networks and referred to as vitrimers. In this study, composite plates were made using vitrimer resin as the matrix material and continuous unidirectional flax fibre fabrics as the reinforcement. A specific early-stage composite part production method is proposed to make the multi-ply flax/vitrimer composite plate. The development of natural fibre–reinforced vitrimer composites is of clear research interest as a promising approach towards sustainable and recyclable novel material systems. Specimens prepared with all the plies oriented 0° exhibited a 129.4 MPa tensile strength and a 12.4 GPa tensile modulus, indicating a 334% increase in tensile strength when compared to the average value of 29.8 MPa obtained for neat vitrimer specimens and a 1140% improvement in the tensile modulus compared to the 1.0 GPa reached for neat vitrimer. The specimens whose plies were oriented 90° are found to deliver a tensile strength of 12.2 MPa and a 1.3 GPa tensile modulus. Applying the classical composite material micromechanics equation to calculate the 0°-direction tensile modulus demonstrated a good agreement with the experimentally obtained value—a 9.6% difference was discovered. Proper fibre/matrix interfacial adhesion was detected when the flax/vitrimer specimens’ surfaces after fracture were examined under scanning electron microscope. The research findings on tensile mechanical properties reveal that the observed flax/vitrimer composites may be potential candidates for replacing typical synthetic fibre–reinforced materials rated for automotive applications and intended for in-plane loaded parts, particularly some inner-body vehicle elements.

## 1. Introduction

Polymer matrix composites are continuously being investigated among material science researchers developing new classes of composite materials aiming to answer today’s and future demands dictated by different application-driven industries. In the race to develop cutting-edge polymer materials and products, designers and engineers need definitive, application-specific characterization to validate material reliability [1]. Analytical techniques such as thermal analysis, rheology, and mechanical analysis are commonly used to characterize the composition, behaviour and physical properties of polymer composite materials [2].

In 2011, Leibler et al. [3] reported a new class of polymers, which were named *vitrimers*, as their viscosity–temperature relationship resembles that of vitreous silica, following an Arrhenius-like dependence, and since then, a considerable increase in the number of vitrimer-related studies reported in the literature has been observed [4,5]. Vitrimers combine the performance of traditional thermosets with the versatility of thermoplastics—having associative dynamic networks, they behave like traditional thermoset resins at service temperatures due to their frozen network topology, while under certain stimuli, such as heat or light, they are able to reorganize their networks while maintaining a constant number of chemical bonds [6]. They can be synthesized from different sources, even bioderived. The vitrimer (imine-linked) resins were observed to be good candidates for producing composites with natural fibres, particularly flax, due to their high interfacial shear strength values [7]. The repair performance of vitrimer resin combined with aligned discontinuous flax fibres has been examined, and these composites showed the potential for a tensile strength recovery of ~50–70% [7], allowing them to be used over several life cycles and therefore contributing to the circular economy. Epoxy-based vitrimers exhibit exceptional self-healing capabilities, allowing them to autonomously repair micro-cracks and damage, restoring mechanical properties [8]. Likewise, vitrimer composite specimens consisting of polycarbonate as a matrix material and natural cellulose paper as a reinforcing framework reached a tensile strength of up to 43 MPa as synthesised and up to 37 MPa after healing [9].

Plant fibres are becoming increasingly common as reinforcements in polymer composites, and one of the main reasons for the increasing interest in plant fibres is their low density (1.25–1.50 g/cm^3^) as compared to the density of glass fibres (about 2.6 g/cm^3^) [10]. The reinforcement used for this study is manufactured from technical flax fibres, and its chemical composition is expected to fall within the ranges reported in the literature for structural flax bast fibres. Therefore, the cellulose content of this particular dry flax fibre fabric is likely to be toward the higher end of typical flax ranges, commonly around 60–80% by weight [11,12], making it easily recyclable as cellulose is biodegradable by many microorganisms [13]. More specifically, the reinforcement adopted for the study is produced from bundles of elementary flax fibres twisted/spun together into continuous yarns and then converted into unidirectional (UD) reinforcement. However, natural fibres have some disadvantages, including poor wettability, poor fibre/matrix adhesion, high moisture absorption (leads to fibre swelling), lower durability, and high variability of its properties [10,14,15]. Amongst the various plant fibres, flax has high mechanical properties, comparable to glass fibres in terms of specific stiffness, as well as high vibration absorption capability [10,16]. One of the major areas seeing growing usage of natural fibre–reinforced composites can be found in the automotive sector, particularly for interior applications—for instance, door panels, seatbacks, and instrument panels [10,17]—and even for exterior parts, with successful examples of a full natural-fibre body kit presented [18,19].

A common challenge is faced by industries across the globe: the need for materials that can meet the complex demands of modern applications while contributing to sustainability goals [20]. It is clear that sustainability requests from OEMs are increasing [21]. In line with that, owing to their numerous advantages, including reprocessability, reparability, and recyclability, the development of vitrimer composites is continually growing [22]. However, compared to traditional polymer matrix composites, most vitrimers have unique processing requirements depending on their dynamic chemistry, which, in turn, limits their widespread application [23]. Therefore, one of the main objectives of this study was to investigate whether the proposed small-scale composite part production method is suitable for obtaining the multi-ply flax/vitrimer composite plates, while meeting adequate mechanical properties. Further, for better fibre impregnation and minimal void content, vitrimeric resins can be heated at any stage of production [24].

Compression moulding is one of the most common processing techniques for fabricating polymeric composite products, and since the early 1950s, this process has seen a remarkable growth in automotive applications by virtue of the development of sheet moulding compounds (SMCs) [25]. Although this process of obtaining the composite parts provides various advantages, like excellent part reproducibility and controllability of the fibre content, SMCs imply the use of synthetic fibres, most often glass fibres embedded as reinforcements into the polyester-based matrix. Despite this, compression-moulded SMC parts may still contain a wide variety of surface and internal defects (e.g., surface pinholes and internal voids, blisters, fibre separation, fibre buckling, etc.) [26]. Typical static mechanical properties for the most common forms of the SMC materials used in automotives, containing randomly oriented chopped E-glass fibres, are range from around 80 to 160 MPa for tensile strength, while tensile modulus values are around 13–16 GPa [26,27]. On the other hand, epoxy-based composites’ production may involve some specific processing requirements, but even with natural fibres as reinforcements, these composites can be proper candidates to substitute conventional SMCs based on their mechanical performances. For instance, Goutianos et al. [28] examined unidirectional (UD) warp knitted flax fabric/epoxy composites obtained using the hand lay-up technique with consolidation under vacuum (Vf ≈ 0.28) and reported reaching a 160 MPa tensile strength and a 15 GPa tensile modulus.

Still, there remains the question of developing fully recyclable composites aligned with circular economy principles and with the flexibility to meet evolving regulatory requirements. This is why vitrimer-based composites reinforced with natural fibres can be conceivably considered as sustainable material alternatives for some automotive applications. In particular, the UD flax fibre–reinforced vitrimer composite examined throughout this study might be interesting for its in-plane mechanical performance. The regulatory requirements referred to herein include the Sustainability Development Goals [29] together with the legal framework of the Corporative Sustainability Reporting Directive [30], which promote the development of lightweight materials with reduced environmental impact. In the automotive sector particularly, these requirements are additionally strengthened by the Directive on End-of-Life Vehicles [31], which builds on and replaces two existing directives: Directive 2000/53/EC [32] on end-of-life vehicles and Directive 2005/64/EC [33] with regard to their reusability, recyclability, and recoverability.

Interior components in automotives, such as door panels, centre consoles, pillar trims, seat backs, and package trays, must typically satisfy requirements in a number of categories. In the present initial study, however, the authors primarily focus on the mechanical performances, particularly the tensile properties. The key challenge associated with the adoption of natural fibre–reinforced composites in automotive applications is that such materials must simultaneously satisfy requirements related to flammability, impact resistance, dimensional stability, volatile organic compound limits, moisture sensitivity constraints, and long-term ageing behaviour. Consequently, qualifying as a (partly) eco-friendly composite for automotive industry is considerably more demanding than merely achieving favourable tensile properties. When focusing specifically on the in-plane mechanical properties of automotive-rated composites, it should be noted that many interior semi-structural parts are predominantly subjected to membrane tension/compression, in-plane bending, shear transfer, and vibration-induced stresses. For thin plate-like structures, insufficient in-plane stiffness can therefore become a critical limitation. This is one of the reasons why flax fibre–reinforced composites remain attractive, as flax fibres can provide relatively high specific stiffness. Although automotive interior components generally do not require aerospace-level mechanical properties, materials commonly need to satisfy approximate ranges of 50–100 MPa (and above) for tensile strength and 2–12 GPa (and above) for tensile modulus, while preferably maintaining densities below approximately 1.5 g/cm^3^ (see also [34,35]).

In this study, specific natural fibre–reinforced polymeric composite samples are thoroughly presented and experimentally characterized in terms of tensile mechanical properties for this initial development stage. The underlying idea is to eventually bring forward the application potential of the proposed flax/vitrimer composite material, based on the study-established findings and expanded by ongoing research into the examined material system. The matrix phase is the recently introduced vitrimer resin comprising recyclable platform chemistry based on the dynamically exchangeable imine-linked polymer networks. It is combined with a reinforcement-grade unidirectional flax fibre fabric with the idea of supporting the development of a novel sustainability-oriented composite material. Although the scope of the present work is limited to tensile mechanical characterization, the findings are intended to contribute to the assessment of the material’s application potential, particularly as replacements for synthetic fibre–reinforced materials used in automotive interiors.

## 2. Materials and Methods

### 2.1. Materials Used for the Composite Plate Constitution

**Figure 1:**
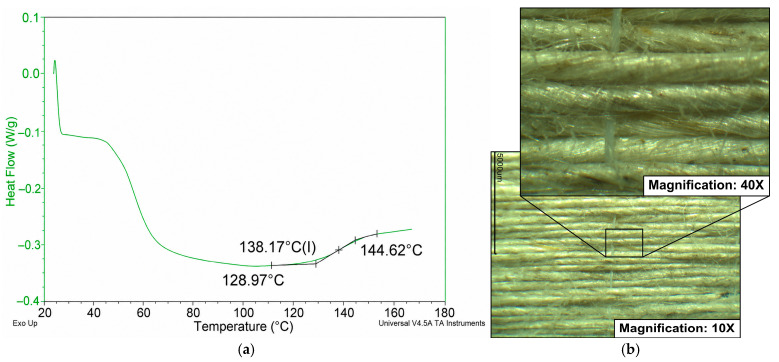
(**a**) DSC thermogram of the vitrimer resin showing a baseline variation (a step-like shift) in the heat flow, with the onset, inflection and endset temperatures indicated—straight lines representing the analysis constructs used for an inflection point determination, and the “+” symbol denoting a signal reported as an inflection point. (**b**) Surface micrograph of the virgin unidirectional flax fibre reinforcement used in the present study.

The vitrimer resin particularly opted for in this study is the *Vitrimax VHM* acquired from *Mallinda Inc*. (Denver, CO, USA). It is capable of being reprocessed even after cure, when heated above the glass transition temperature ($T_{g}$). There is a wide range of useful $T_{g}$s that this resin can be cured to (80–180 °C), and as such, it is appropriate for use in various applications including automotive industry [36]. Differential scanning calorimetry (DSC) measurements were performed for the present study using a *DSC Q10* from *TA Instruments* (New Castle, DE, USA) over a temperature range of 25 °C to 170 °C under a nitrogen atmosphere, at a scan rate of 10 °C min^−1^. The temperature of 138 °C was determined as the inflection point during the first heating cycle and may be identified as the glass transition temperature (Figure 1a). The inflection point was observed at 138 °C, which exceeds the nominal average value of 130 °C based on the $T_{g}$ range given by the resin manufacturer, suggesting that a high degree of polymer network cross-linking may have been achieved. It should be noted that the observed baseline variation in the exothermic direction cannot be unambiguously attributed to the glass transition and that subsequent dynamic mechanical analysis (DMA) measurements would enable a more reliable determination of $T_{g}$, as mechanical loss factors (tan δ peaks) generally provide a more sensitive indicator of $T_{g}$ than DSC measurement alone. The natural fibre fabric used as a reinforcement in the studied composite material is the non-crimp unidirectional flax fabric (*ampliTex™ 5025*), commercially available from *Bcomp Ltd.* (Fribourg, Switzerland). The *ampliTex™* provides a range of natural fibre reinforcements for composites. This reinforcement fabric’s architecture is stated to be engineered to offer high performance at low weight, with minimal impact on the environment, while being compatible with different composite processing methods like vacuum infusion, wet layup, resin transfer moulding, autoclave processes, and compression moulding [37]. The surface micrograph of the virgin flax fabric reinforcement was recorded using the *DM-143-FBLED* digital stereo microscope from *Motic* (Hong Kong, China) and is presented in Figure 1b. It can be observed that the flax fabric reinforcement utilised for this study consists of elementary flax fibres assembled into bundles and processed into continuous yarns oriented along a single direction, corresponding to the principal reinforcement direction. The general properties known for the isolated reinforcement and those of the matrix material used in this study are outlined in Table 1.

### 2.2. Composite Plate Specimens’ Fabrication and Characterization Methods

To make the multi-ply flax/vitrimer composite plate in-house, a small-scale composite part production method is proposed. It is a combination of wet layup accompanied by heat and pressure application throughout the preparation of individual plies, followed by vacuum bagging the whole stack-up of plies and curing under vacuum. The process is discussed in more detail in the following.

#### 2.2.1. Preparation Steps

**Figure 2:**
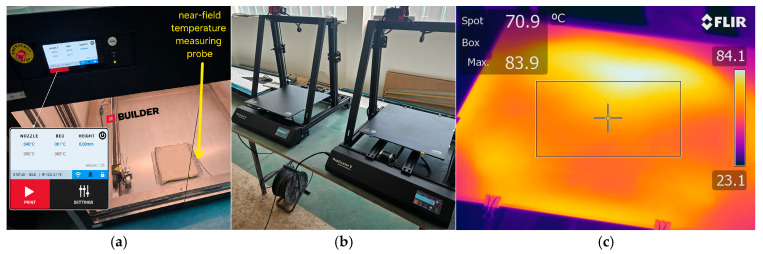
(**a**) Flax fibres drying prior to impregnation with vitrimer matrix. (**b**) 3D printers with heated plates appropriate for custom composite ply preparation. (**c**) Thermal image taken to confirm near homogeneous heat field over the used heated plate.

Prior to flax fibre fabric impregnation with vitrimeric resin, the fibres were effectively dried at ~50 °C for 1 h 30 min in a closed heated chamber. The large-scale 3D printer *Builder Extreme 3000 PRO* from *Builder 3D Printers B.V.* (Noordwijkerhout, The Netherlands) served this purpose (Figure 2a). Since vitrimer resin manipulation requires heat when mixed to maintain a workable viscosity (see also [38] for the rheology plot of a similar vitrimer), but also a certain amount of pressure application for proper fibre impregnation, there emerged the need for a heated press. There was no such resource available in-house, so the authors opted for a custom solution entailing heated beds from two 3D printers with appropriate heated plate dimensions—one plate to serve as the base work area for lamination, and an upper one with a weight on it to simulate the heated press effect. Two available desktop 3D printers (*Duplicator 9* from Jinhua Wanhao Spare Parts Co., Ltd., Jinhua, China*—*see Figure 2b) with great build volumes were used for this purpose, providing two heated plates with dimensions of 400 × 400 mm, making them perfectly suitable for the intended 300 × 300 mm composite plate fabrication. To ensure near-homogeneous heat field over the 3D printers’ heated plates, thermal images were taken (Figure 2c) using a *FLIR T620* camera from *FLIR Systems AB* (Stockholm, Sweden). The target working table (plate) temperature of ~80 °C was therefore confirmed.

Primarily for use as the tooling table for the stack-up of plies after individual plies are impregnated, an aluminium plate AlMg4,5Mn (AL 5083) was procured, with dimensions of 350 × 350 × 15 mm, two surfaces precision milled (roughness Ra 0.4 μm), and foiled on both sides. It was also used to apply pressure to the upper heated plate throughout the process of impregnating individual plies (will be discussed in detail in the following section). Apart from that, specific consumables for the vacuum bagging process of the final multi-ply flax/vitrimer stack-up also needed to be procured, conforming with high curing temperature requirements. Therefore, the vacuum bagging materials rated for higher temperatures were obtained to ensure unimpaired composite plate curing in the oven at 180 °C.

#### 2.2.2. Fabrication Process

**Figure 3:**
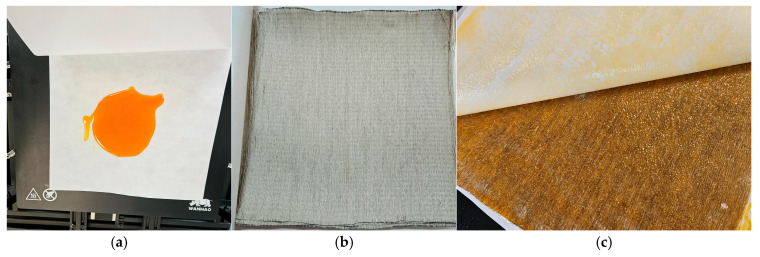
(**a**) Mixed vitrimer prepared for making the matrix film. (**b**) Dry UD flax fibre fabric. (**c**) Flax fabric impregnated with vitrimer matrix (one ply obtained).

**Figure 4:**
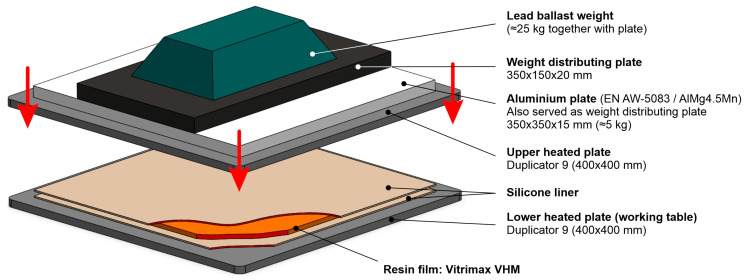
Schematic representation of the processing setup employed during the resin film and individual ply preparation prior to laminate fabrication (the arrows indicate the direction of pressure application).

**Figure 5:**
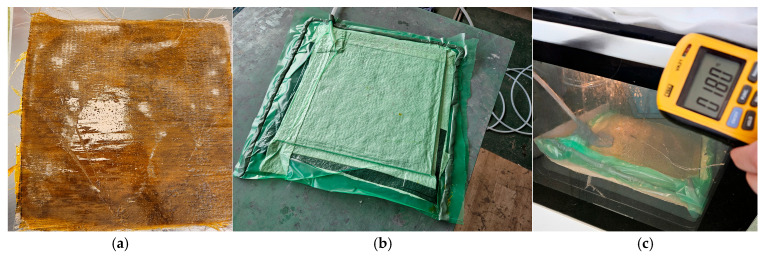
(**a**) Six-ply flax/vitrimer stack-up—all plies in 0° orientation. (**b**) Stack-up of plies, vacuum bagged together with underlying aluminium plate. (**c**) Curing in oven.

**Figure 6:**
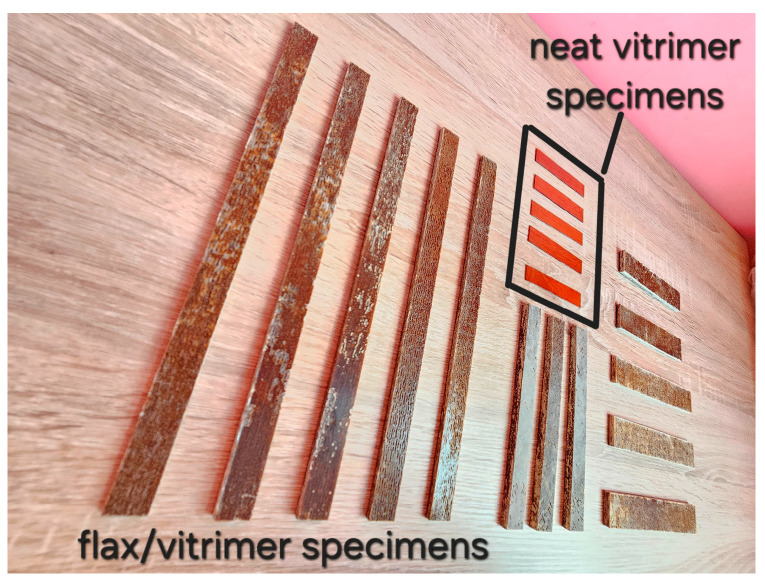
Series of samples for tensile testing.

The process followed to make the multi-ply flax/vitrimer composite plate is described here in detail, step by step:

(1) First, the resin film was created by: (a) mixing the two-part vitrimer resin (imine hardener and epoxy blend) at a 1.5-to-1 ratio; (b) pouring the mixed resin onto the silicone liner placed on the lower 3D printer’s heated plate (Figure 3a); (c) covering with the upper silicone liner part; (d) pressing with the upper heated plate and the weight added to it for ~30 s to make the film. The overall weight applied was approximately 30 kg (around 300 N force), considering the lead ballast placed on top and its weight-distributing plate (total of ~25 kg) together with the previously mentioned aluminium plate (~5 kg), as illustrated in Figure 4. Owing to its stiffness and dimensions, which essentially covered the entire processing area, the aluminium plate additionally contributed to promoting a relatively uniform pressure distribution. It is important to note here that the imine hardener must be pre-heated in an oven at 85 °C for 2 h (in a closed can) to reach the target viscosity that can be preserved along the process, while epoxy blend is stored at room temperature. Before mixing, the imine hardener and the epoxy blend were weighed out on the precision scale to fulfil the required ratio (more on this is outlined in Section 2.2.3). After mixing, in order to maintain the workable viscosity, the mixed resin can was kept in the oven at ~80 °C between the pouring sequences, while the 3D printers’ plates were pre-set to continuous heating at 80 °C and maintained at that temperature the whole time while the individual plies were laminated.
(2) When the vitrimer film was ready, the upper heated plate was removed, and the silicone liner was opened to access the resin film. The 300 mm x 300 mm UD flax fabric (Figure 3b) was promptly laid in between the split resin, and the liner was closed again, now covering the resin and the fabric laid inside it. Right away, the upper heated plate was placed on top to apply pressure for ~1 min.
(3) After pressing between the heated plates, the flax fabric was impregnated with the vitrimer resin (Figure 3c), and this way, one ply of the intended composite plate was obtained. Overall, six flax/vitrimer plies were made via the so-far described process. Until initiating the stacking of plies, one on top of the other, to get a laminate plate form, the impregnated plies (closed within the silicone liners) were stored in a freezer at −10 °C to stop the polymer crosslinking process.
(4) The impregnated plies were brought to room temperature when ready for stacking. The previously mentioned 15 mm-thick aluminium plate was release-coated and prepared as a non-porous tooling surface. Individual plies were laid on the tooling plate (Figure 5a) in the desired orientation—for this study, all six UD plies were placed in the same (0°) orientation—in a stack-up form that would lead to the intended flax–vitrimer composite plate after curing.
(5) Next, the stack-up was packed into the vacuum bag together with the corresponding vacuum bagging materials, and the vacuum was pulled (Figure 5b) and maintained at a pressure differential of ~1 bar with the vacuum pump over the curing time. At this stage and during the upcoming steps, it is important to ensure no leaks.
(6) The vacuum-bagged stack-up was then put into the oven for curing, first at 130 °C for 45 min, then at 180 °C for another 1 h and 30 min (Figure 5c). This curing cycle was applied in accordance with the recommendations provided by the resin manufacturer. It included an extended high-temperature curing stage intended to promote a higher degree of conversion and enhance the thermal stability of the cured network. The curing procedure was performed using an electrically heated oven modified with a PID temperature controller, featuring a power rating of 2600 W and an internal chamber capacity of 51 L. Both upper and lower heating elements were utilized together with forced-air circulation provided by an internal fan to promote uniform heat distribution. The temperature in the oven was monitored continuously. Before debagging the part, the part was left to cool to room temperature under vacuum. Upon debagging, the final flax/vitrimer composite plate was obtained.
(7) Finally, composite specimens for tensile testing (Figure 6) were cut out of the cured flax/vitrimer composite plate on a router table with a 1.4 mm drill bit. The nominal specimens’ dimensions used as a reference for cutting out of the fabricated composite plate were intended to be the ones recommended by the ASTM D3039 [39]—250 mm length and 15 mm width for the specimens cut along the fibre direction (0° unidirectional) and 175 mm length and 25 mm width for the specimens cut perpendicular to the fibre orientation (90° unidirectional). The final dimensions obtained for the two specimen groups are listed in Table 2, with the indicated average cross-section dimensions measured at multiple points along the specimens’ length preparatory to testing.

#### 2.2.3. Fibre Fraction Pre-Calculations

Considering the *Rule of Mixture* (*RoM*) equation $\rho_{c}=\rho_{f}v_{f}+\rho_{m}v_{m}$—where $v_{f}$ and $v_{m}$ are the fibre and matrix percentage fraction of the composite volume; and $\rho_{f}$, $\rho_{m}$, and $\rho_{c}$ are the fibre, matrix, and composite densities, respectively—fibre volume fraction per ply was pre-calculated theoretically, with basic assumptions of the classical composite material micromechanics. Since fibre/matrix mass ratio per ply was measured precisely when laminating, adding the mass equation $m_{c}=m_{f}+m_{m}$ to the aforementioned rule of mixture results in obtaining the theoretical volumetric fractions, given that $m_{f}=\rho_{f}V_{f}$ and $m_{m}=\rho_{m}V_{m}$, where $m_{f}$ and $m_{m}$ are the mass values of the fibre and matrix, respectively, and $V_{f}$ and $V_{m}$ are their volumes.

Applying the 63.5 g (on average) of mixed vitrimer resin (the matrix material) and ~24.75 g (275 g/m^2^ × 0.3 m × 0.3 m) of flax fibre fabric per ply, and using the densities outlined in Table 1, the fibre/matrix volume percentage ratio was calculated to be 23%/77%. This is to be utilised as a theoretically anticipated baseline for the comparison with the experimentally obtained modulus of elasticity values. A study addressing aligned discontinuous flax fibre preforms combined with a similar vitrimer matrix reported calculating the fibre volume fraction of 17.9% based on the *RoM* model [7]. The simplicity of the *RoM* model implies that it has become a widely used model within continuous fibre–reinforced polymers for basic pre-calculations of the material properties along the fibre direction, such as the 0° modulus of elasticity ($E_{1}=E_{f}v_{f}+E_{m}v_{m}$). If a more detailed analytical model is to be applied, several modification factors should be accounted for, such as the fibre length efficiency factor, the reinforcement orientation distribution factor, the fibre diameter distribution factor, and the fibre area correction factor used to account for fibre area measurement discrepancies, especially when natural fibres are used as reinforcements [12]. Also, it is important to note that the fibre volume fraction of 23% reported in the present study does not account for the effects that are influential later in the process of obtaining the composite laminate, such as resin loss during the laminate consolidation stage. Consequently, the reported value should be regarded as an estimate and interpreted with appropriate caution. To assess the composite material’s exact stiffness as of the finally obtained plate-like part, experimental testing was conducted.

#### 2.2.4. Methods of Experimental Testing Performed

A universal testing machine together with a strain indicating device was utilised to perform the uniaxial tensile testing in accordance with the ASTM D3039 standard. The gauge length applied when tested the 0° UD specimens was 50 mm, and the one practiced for the 90° UD specimens was 25 mm. The tensile modulus was calculated as the slope of the stress–strain curve within the strain range of 500 με to 2000 με. The 0° unidirectional specimens in general are the most user-sensitive configuration, since the slightest fibre/load misalignment, which can easily occur due to either specimen preparation or testing problems, or both, can reduce the output strength value significantly [40]. Prior to testing, specimens’ side edges were lightly polished with fine abrasive paper to eliminate machining defects and minimise edge-initiated stress concentrations. Tabs made of the same material as is being tested were used to facilitate gradual load transfer from the grips to the test specimens. It should be noted that the design of mechanical test coupons, especially those using end tabs, remains to a large extent an art rather than a science, with no industry consensus on how to approach the engineering of the gripping interface [39].

Scanning electron microscopy (SEM) was used following the tensile testing to examine the fracture surfaces of the tested specimens. Prior to SEM analysis, the samples were mounted on the aluminium stubs using conductive carbon adhesive tape and sputter-coated with a thin Au–Pd layer using a *Polaron SC502* (Fison Instruments, Glasgow, UK) sputter coater. SEM micrographs were acquired at magnifications of 500× and 5000× to evaluate the overall surface morphology and finer microstructural features, respectively.

The properties that this study aimed to hereby evaluate were the maximum normal stress (tensile strength), the strain at failure or strain at reaching the tensile strength if different, the tensile modulus (stiffness), and the quality of matrix adhering to fibre surfaces. These properties are to be studied for three sample series: the 0° UD flax/vitrimer, the 90° UD flax/vitrimer, and the neat vitrimer specimens.

## 3. Experimental Testing and Results

### 3.1. Experimental Setup

Uniaxial tensile testing was carried out for the prepared flax/vitrimer specimens on the *Shimadzu AGS-X* universal testing machine (Shimadzu Europa GmbH, Duisburg, Germany) to the breaking point. The neat vitrimer specimens were also subjected to tensile testing. Normal strain data along the loading direction were gathered using the *Epsilon 50 mm* clip-on extensometer (Epsilon Technology Corp., Jackson, WY, USA). Testing was conducted at room temperature with the crosshead speed set to 2 mm/min. A series of five samples were prepared. Load (force) data was recorded by the testing machine’s built-in load cell sensor, while displacements of gauge section points were measured by applying the clip-on linear extensometer. The surface morphology of the samples was examined using a *TESCAN MIRA3 FEG* (Tescan, Brno, Czech Republic) field emission scanning electron microscope (FE-SEM) operated at an accelerating voltage of 20 kV.

### 3.2. Tensile Testing Results and Discussion

**Figure 7:**
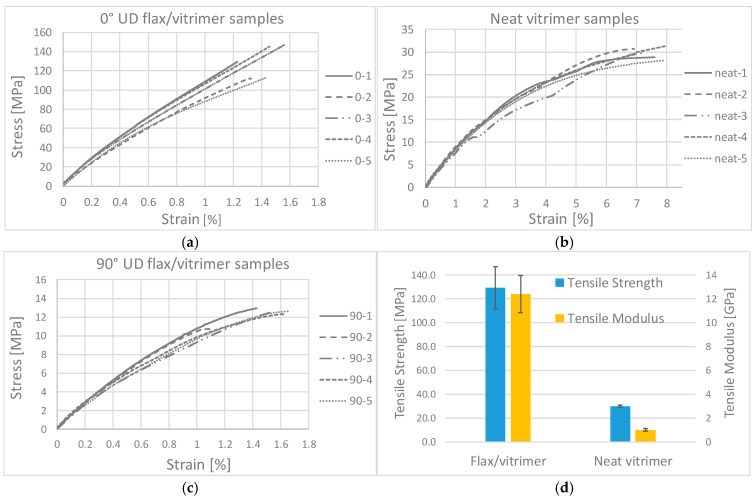
Stress–strain curves obtained for: (**a**) 0°-oriented UD flax/vitrimer composite, (**b**) 90°-oriented UD flax/vitrimer composite, (**c**) neat vitrimer. (**d**) Comparison of the tensile strength and tensile modulus values between the 0° UD flax/vitrimer composite and the neat vitrimer.

It was found that the 0° UD flax/vitrimer specimens exhibit a tensile strength of 129.4 MPa (Figure 7a) and a longitudinal tensile modulus ($E_{1}$) of 12.4 GPa, according to the ASTM D3039 guidelines for calculation. This means there was a 334% increase in tensile strength when compared to the average value of 29.8 MPa obtained for the neat vitrimer specimens (Figure 7b) and a 1140% improvement in the tensile modulus, compared to 1.0 GPa reached for the neat vitrimer (see Figure 7d).

As expected, the 90° UD flax/vitrimer samples are an order of magnitude weaker than the 0° UD samples due to fibres as the reinforcing part presumably being oriented strictly perpendicular to the loading direction (later confirmed via SEM). These lower values were surveyed both for the tensile strength and the tensile modulus. The 90° UD samples were found to have an even lower tensile strength than the neat vitrimer samples, reaching an average value of only 12.2 MPa (see Figure 7c). This behaviour may essentially be attributed to the fibres’ orientation being perpendicular to the loading direction, whereby the fibres contribute negligibly to the load-carrying and may instead adversely affect the matrix-dominant response in that direction. In addition, the incorporation of fibres during lamination might have introduced local inhomogeneities into the vitrimer matrix. These features could possibly reduce the effective load-bearing cross-section and thereby lower the maximum stress the final part can withstand. Nevertheless, the 90° UD samples’ tensile stiffness assessed experimentally is 30% higher than that derived for the neat vitrimer samples, as a value of 1.3 GPa was calculated for the transverse tensile modulus ($E_{2}$).

The investigated tensile mechanical properties of all the tested sample series are summarised in Table 3. The complete set of experimentally determined values is given for the tensile strength and the extensional strain when the tensile strength value was reached, together with the calculated tensile modulus values for the observed direction. The average values obtained for these material properties are accompanied by standard deviation and relative standard deviation values. The measurement deviations can be considered acceptable and consistent with the inherent variability commonly reported for natural fibre–reinforced composites (e.g., see the results given by [7,41]), indicating good repeatability overall.

The failure of all the composite samples tested occurred abruptly right after reaching the maximum normal stress value. As for the failure mode, the 0° UD samples predominantly fractured at an angle and starting in the vicinity of the grip (used tabs) area, while the 90° UD samples’ failure was lateral (along the fibres) and entirely within the gauge section with no need for tabs. The latter is in accordance with [42], stating that untabbed specimens may be acceptable for lower-strength composites such as cross-ply laminates (with 90° plies on the outer surfaces) and textile composites. Compared to conventional epoxy systems, the neat vitrimer specimens exhibited higher elongation-at-break values, indicating enhanced polymer network mobility.

The typical tensile mechanical properties for the most common forms of the SMC materials used in automotive applications are mentioned in the introduction section. When the present study findings on tensile strength and stiffness properties are compared to these values, the observed flax/vitrimer composite may prospectively be considered as a candidate for replacing conventional synthetic SMC materials, taking into account that additional investigations concerning the requirements—regarding load transfer capability and prospectively regarding damage tolerance—outlined in the introduction section are still necessary. In this sense, the evaluated results suggest potential applicability in lightweight and composite-intensive automotive structures under service loadings—e.g., door panels (inner structural inserts), roof panels, pillar cover panels, and seat supports, in conformity with circular economy initiatives and vehicle end-of-life directives.

It should be highlighted that the intention of the present study is not to evaluate a specific laminate design against predefined automotive application requirements, but rather to establish the tensile strength and stiffness performance of the proposed material system. A comprehensive evaluation would require additional investigations of relevant mechanical properties, including shear, flexural, impact, fatigue, and creep behaviour, together with consideration of the service conditions and loading scenarios associated with the intended application. The results reported in the present work correspond to unidirectional composites, which inherently exhibit highly anisotropic mechanical behaviour, whereas in engineering practice, such anisotropy is commonly managed through the stacking of plies with different fibre orientations to achieve the required balance of mechanical properties. Balanced symmetric laminate configurations are often employed in automotive components to improve dimensional stability and mitigate extension-twisting coupling effects. However, the design and optimization of laminate stacking sequences were beyond the scope of the present study. Consequently, the effective in-plane properties of a realistic laminate configuration would be expected to differ from those measured in the unidirectional laminate investigated herein.

### 3.3. SEM Image Analysis

**Figure 8:**
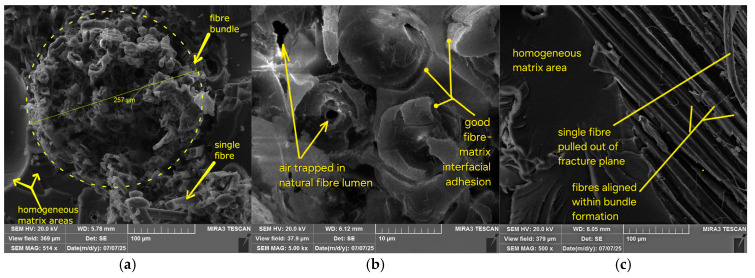
(**a**) SEM image of the 0° orientation flax/vitrimer specimen fracture surface with a fibre bundle in the cross-section highlighted. (**b**) Higher-magnification (5000×) SEM image of the 0° orientation flax/vitrimer specimen showing single fibre/matrix interfacial bonding. (**c**) Fracture surface of the specimen whose plies are oriented at 90°, with the captured fibres lying within the image plane.

Looking at Figure 8a, the fibres appear to be properly wetted and well adhered to the vitrimer matrix, with visible homogeneous matrix areas. The displayed example shows the 0° orientation specimen, where the fibres point out of the fracture surface plane. It can be seen that the fibres are distributed throughout the cross-section part in a mainly good order, certainly contributing to fairly uniform load-carrying among the fibres caught within the observed cross-section—what cannot be seen are the areas of fibre accumulation with poor wettability or apparent great areas of sole matrix material with no fibres at all where expected. However, a qualitative comparison between the scan shown in Figure 8a and the scanning electron micrographs of the aligned flax fibre–reinforced vitrimer specimens examined by Kandemir et al. [7] suggests that those specimens reported by Kandemir et al. may exhibit morphological features consistent with a higher degree of laminate consolidation. Here, consolidation refers to the effectiveness of laminate compaction during processing. The two composite systems differ in composition and processing conditions, so the conclusions should be drawn with caution and not directly applied. Nevertheless, the observed difference might be associated with the higher pressure applied in the second stage of the curing process reported in [7], as increased processing pressure is commonly recognized as a factor promoting laminate consolidation. The fibre aggregation highlighted in the figure is actually one bundle of single flax fibres combined to form a joined load-carrying unit in the UD flax fabric. As such, the single fibres are actually arranged within bundles that seem to be distributed in relatively good order across the visible specimen cross-section, as referred to earlier. Figure 8b, a higher-magnification image (5000×), clearly displays good matrix adhesion all around the single fibres’ diameter, with certain voids present in the fibre internal cavity (lumen) that are inevitable as an intrinsic feature of natural fibres. Other than that, there are no notable voids or other kinds of material inhomogeneities present in the observed image. This observation provides support for the adequacy of the proposed UD flax/vitrimer composite fabrication method in producing plate-like parts with reasonably consistent tensile load-bearing capability. It could be regarded as additional support for interpreting the afore-presented tensile strength and stiffness properties as comparable to those of the synthetic fibre–reinforced counterparts that are still vastly used in automotives.

The cross-sectional morphology of the elementary flax fibres observed in the SEM micrograph shown in Figure 8b is consistent with that reported in studies on flax fibres and flax fibre–reinforced composites (e.g., [10,41]). In composite materials, flax fibres are frequently observed as closely packed aggregates, where the boundaries of individual fibres may partially conform to those of neighbouring fibres (e.g., [38]). Consequently, the precise cross-sectional shape of individual fibres cannot always be unambiguously distinguished from the micrograph and may appear less clearly defined than for isolated fibres. The diameters of the elementary flax fibres observed in Figure 8b are approximately 10–20 μm, which agrees well with the ranges commonly reported in the relevant literature.

Figure 8c brings forward the 90° specimen fracture surface after tensile testing, with distinct areas of matrix material (left) and fibres (right). The fibres caught within the displayed cross-section confirm fairly even distribution and alignment within the bundle in this plane as well, together with the homogeneous solely matrix material part, without clear air gaps captured. This proper fibre arrangement confirms that the intended stacking sequence was successfully applied when fabricating the specimens and is consistent with the substantially lower tensile strength and stiffness values obtained for the 90° specimens—because the fibres as load-bearing members are verified to be in fact perpendicular to the loading direction while tensile testing the final part. The observed specimen fractured along the plane incorporating the fibre bundles, which can be seen in the fact that the single fibres of the captured bundle lie within the fracture surface (mainly staying incorporated within the matrix), with one single fibre clearly pulled out of the fracture plane. The predominance of fibres remaining embedded within the matrix, together with the limited occurrence of fibre pull-out, supports the presence of adequate fibre/matrix interfacial adhesion. Furthermore, no prominent voids were observed within the examined region, suggesting an acceptable degree of laminate consolidation under the processing conditions employed.

### 3.4. Comparison with Theoretical Results for $E_{1}$ and $E_{2}$

Bearing in mind the definition of the composite material, it is clear that the material elastic constants directly correspond to the characteristics of the fibres used as reinforcements, the matrix material characteristics, and the fibre/matrix volume fraction. Thus, the composite material elastic modulus values are explicitly dependent on the fibre and the matrix elastic moduli ($E_{f}$, $E_{m}$). As such, the longitudinal elastic modulus can theoretically be determined using the *Rule of Mixture* (*RoM*) equation already given in Section 2.2.3. Applied to the here-studied 0° UD flax/vitrimer composite, respecting the given value of $E_{f}=$ 56.0 GPa [37] and the value of $E_{m}=$ 1.0 GPa obtained for neat vitrimer, the theoretical result for the longitudinal elastic modulus is $E_{1}=$ 13.7 GPa. This means there is a 9.6% difference when compared to the experimentally obtained value of 12.4 GPa. This observation is in compliance with the general guideline from composite materials textbooks stating that the *Rule of Mixtures* provides a reliable estimate for the longitudinal elastic modulus of continuous fibre–reinforced composites due to the iso-strain (Voigt model) condition along the fibres [43]. However, *RoM* generally fails to accurately predict the transverse modulus of elasticity $E_{2}$ (usually underestimates the experimental value), where matrix-dominated behaviour, fibre–matrix interaction, and stress concentrations necessitate more-advanced micromechanical models. Despite that, when applied particularly to the here-studied 90° UD flax/vitrimer composite, the Reuss model, $1/E_{2}\;=v_{f}/E_{f}\;+v_{m}/E_{m}\;$ (also known as the *Inverse Rule of Mixtures*), actually provided a near-perfect estimate of the transverse elastic modulus, resulting in just a 0.6% difference compared to the experimentally assessed value of 1.3 GPa. This was expected to some extent, given the relatively low fibre volume fraction, as well as the inherent variability of natural fibres. It should be noted that the *Rule of Mixtures* relies on the assumptions of ideal fibre alignment and flawless fibre/matrix interfacial bonding, both of which are challenging to realize in natural fibre composites. Again, it is important to emphasize that the mechanical property estimates obtained using the *RoM* model are highly sensitive to the input fibre volume fraction value. Therefore, if such estimates are intended to form a basis for the principal study findings, rather than serve solely for comparison purposes as in the present study, a more rigorous determination of this parameter would be required, preferably through direct experimental measurement.

## 4. Conclusions and Future Work

The examined vitrimer-based composite material reinforced with continuous unidirectional flax fibre fabric is found to be suitable for producing via the presented small-scale method for the commencing research, as the obtained plate-like parts reached competent mechanical performance so far regarding the tensile mechanical properties. The efficiency of the proposed early-stage fabrication method is supported by SEM observations of the specimens’ fracture surfaces, indicating adequate fibre/matrix interfacial adhesion. Tensile strength values for the 0° orientation UD flax fibre–reinforced vitrimer material, experimentally assessed in accordance with the ASTM D3039, revealed a more than 4.3-fold increase when compared to the values obtained for the neat vitrimer material, while tensile stiffness showed a 12.4-fold improvement. The 0° unidirectional flax/vitrimer specimens reached an average value of 129.4 MPa for the tensile strength and 12.4 GPa for the tensile modulus. The 90° UD flax/vitrimer samples exhibited strength and stiffness values that were an order of magnitude lower than the ones oriented 0°, which can be attributed to the fibres being oriented perpendicular to the loading direction. SEM observations confirmed this fibre orientation in the final plate part, thereby supporting the stacking accuracy during fabrication. Comparison of the experimental results with the estimates given by basic composite material micromechanics models demonstrated reliable agreement for both the longitudinal and even the transverse tensile modulus. Although additional testing is needed, evaluated research findings on tensile mechanical properties of the studied flax/vitrimer composite suggest that this sustainability-oriented material could be a potential candidate for replacing typical synthetic fibre–reinforced SMC materials intended for in-plane loaded parts, particularly for automotive inner-body elements.

Since vitrimers are able to readily flow above a characteristic temperature and thus behave like a viscoelastic re-shapeable fluid, flax/vitrimer composites might also be interesting to examine upon exposure to the appropriate heat stimuli and study in terms of their self-healing capabilities and the efficiency of regaining their initial mechanical properties after damage. Therefore, a specific part of conducting future tests shall be dedicated to investigating the mechanical performance of the test specimens upon damage and after activating the vitrimer repair mechanism.

## Figures and Tables

**Table 1:** Properties for the original constituents of the composite material being developed [36,37].

**Matrix Material**	**Matrix—** **Polymer Type**	**Mix Ratio** **Imine Hardener:Epoxy Blend**	**Mixing Conditions**	**Cured Resin Density [g/cm^3^]**
Polymer	Vitrimer	1.5:1	Pre-heating the imine hardener	1.15
**Reinforce-ment**	**Weave**	**Areal weight [g/m^2^]**	**Uncompressed thickness [mm]**	**Density [g/cm^3^]**
Flax fibres	UD fabric	275 ± 5%	0.57 ± 0.04	1.50

**Table 2:** Dimensions of prepared flax/vitrimer specimens.

Specimen	Nominal Dimensions		Measured Cross-Section Dimensions	
	Length [mm]	Width [mm]	Avg. Thickness [mm]	Avg. Width [mm]
0°—1	250	15	3.93	15.48
0°—2	250	15	3.37	14.89
0°—3	250	15	4.30	14.66
0°—4	250	15	4.52	15.53
0°—5	250	15	4.54	15.44
90°—1	150	25	4.65	25.33
90°—2	150	25	4.53	25.41
90°—3	150	25	4.31	25.49
90°—4	150	25	4.66	25.41
90°—5	150	25	4.08	25.48

**Table 3:** Summarised values for the tensile mechanical properties evaluated in the study.

Sample Series	Tensile Strength [MPa]			Strain [%] at Tensile Strength			Tensile Modulus [GPa]		
	Average	St. Dev.	Rel. St. Dev. [%]	Average	St. Dev.	Rel. St. Dev. [%]	Average	St. Dev.	Rel. St. Dev. [%]
0° UD flax/vitrimer	129.4	16.7	12.9	1.40	0.13	9.2	12.4	1.1	9.1
90° UD flax/vitrimer	12.2	0.8	6.9	1.46	0.24	16.5	1.3	0.0	3.2
neat vitrimer	29.8	1.3	4.4	7.52	0.47	6.3	1.0	0.1	7.9

## Data Availability

The raw data supporting the conclusions of this article will be made available by the authors on request.
